# Wāhine hauora: linking local hospital and national health information datasets to explore maternal risk factors and obstetric outcomes of New Zealand Māori and non-Māori women in relation to infant respiratory admissions and timely immunisations

**DOI:** 10.1186/1471-2393-13-145

**Published:** 2013-07-10

**Authors:** Sara Filoche, Susan Garrett, James Stanley, Sally Rose, Bridget Robson, C Raina Elley, Bev Lawton

**Affiliations:** 1 Women’s Health Research Centre, Dept of Primary Health Care and General Practice, University of Otago, Wellington, New Zealand; 2 Dean’s Department, University of Otago, Wellington, New Zealand; 3 Te Rōpū Rangahau Hauora a Eru Pōmare, University of Otago, Wellington, New Zealand; 4 School of Population Health, University of Auckland, University of Otago, Wellington, New Zealand

**Keywords:** Health disparities, Indigenous health, Routinely collected health data, Health information datasets, Maternity care, Infant health, Hospital admissions, Respiratory diseases, Immunisation

## Abstract

**Background:**

Significant health inequities exist around maternal and infant health for Māori, the indigenous people of New Zealand. The infants of Māori are more likely to die in their first year of life and also have higher rates of hospital admission for respiratory illnesses, with the greatest burden of morbidity being due to bronchiolitis in those under one year of age. Timely immunisations can prevent some respiratory related hospitalisations, although for Māori, the proportion of infants with age appropriate immunisations are lower than for non-Māori. This paper describes the protocol for a retrospective cohort study that linked local hospital and national health information datasets to explore maternal risk factors and obstetric outcomes in relation to respiratory admissions and timely immunisations for infants of Māori and non-Māori women.

**Methods/Design:**

The study population included pregnant women who gave birth in hospital in one region of New Zealand between 1995 and 2009. Routinely collected local hospital data were linked via a unique identifier (National Health Index number) to national health information databases to assess rates of post-natal admissions and access to health services for Māori and non-Māori mothers and infants. The two primary outcomes for the study are: 1. The rates of respiratory hospitalisations of infants (≤ 1 yr of age) calculated for infants of both Māori and non-Māori women (for mothers under 20 years of age, and overall) accounting for relationship to parity, maternal age, socioeconomic deprivation index, maternal smoking status. 2. The proportion of infants with age appropriate immunisations at six and 12 months, calculated for both infants born to Māori women and infants born to non-Māori women, accounting for relationship to parity, maternal age, socioeconomic deprivation index, smoking status, and other risk factors.

**Discussion:**

Analysis of a wide range of routinely collected health information in which maternal and infant data are linked will allow us to directly explore the relationship between key maternal factors and infant health, and provide a greater understanding of the causes of health inequalities that exist between the infants of Māori and non-Māori mothers.

## Background

Health inequities for Māori, the indigenous people of New Zealand, are persistent and significant, and are particularly evident around maternal and infant health [1]. This paper describes the protocol for a retrospective cohort study that links local hospital and national health information datasets to explore maternal risk factors and obstetric outcomes in relation to respiratory admissions and timely immunisations for infants (≤ 1 yr of age) of Māori and non-Māori women.

### Obstetric outcomes in New Zealand

In New Zealand there are approximately 60,000 live births per annum [2]. However, despite international declines in infant death rates over the last 40 years, New Zealand’s infant death rate still remains high at 5.2 deaths per 1,000 live births (ranked 27th of 34 OECD countries for which information is available [3]) New Zealand’s infant mortality rates are lower than the USA (6.1 deaths per 1, 000 live births) but higher than the UK (4.2 deaths per 1, 000 live births) [3]. Furthermore, pregnant or recently pregnant Māori women are more likely to die compared to NZ European women [4], Māori have a higher rate of stillbirth and neonatal death, and Māori infants are more likely to die in their first year of life compared to non-Māori infants [5-7].

### Infant respiratory health in New Zealand

Respiratory diseases are a leading contributor to infant morbidity and mortality [1,8]. During 2002–2006, hospital admissions for lower respiratory tract conditions were highest amongst those under 5 years of age, with the greatest burden of morbidity being due to bronchiolitis admissions in those under 1 year of age [9]. Māori infants have high rates of hospital admission for respiratory illnesses (including acute bronchitis, bronchiolitis, acute upper respiratory tract infections and pneumonia [7]). Nationwide data covering the 2003 to 2005 period has shown that respiratory hospitalisation rates for infants less than 1 year of age were 18,854 per 100,000 for Māori, and 9,214 per 100,000 for non-Māori [7]. Poor access to primary health care, due to factors such as limited finances, is known to contribute to the health inequalities that exist between Māori and non-Māori [7]. Adequate care provided at the primary care level, as well as timely immunisations [10] can prevent some respiratory related hospitalisations [10-13].

### Immunisation coverage in New Zealand

The New Zealand Childhood Immunisation Schedule offers free immunisations protecting against nine vaccine preventable diseases from the age of 6 weeks to 11 years of age for: Diphtheria, Tetanus, Pertussis (whooping cough), Poliomyelitis, Hepatitis B, *Haemophilus influenza* type B, Measles, Mumps and Rubella [14]. Immunisation against these diseases also confers health benefits towards other significant health conditions that are prevalent in New Zealand’s children, including bronchiolitis and bronchiectasis, which are also more prevalent for Māori children, and which are associated with a socioeconomic gradient [9,15]. Although immunisation has the potential to reduce health conditions with a socioeconomic gradient, health disparities continue to be prevalent and the rates of vaccine-preventable diseases are higher in New Zealand compared with other developed countries [9].

Immunisation coverage is measured at the ‘milestone ages’ of 6, 12, 18 and 24 months, and 5 years and 12 years old [16]. If a child has received all of their age appropriate immunisations by the time they have reached the milestone age they are regarded as being fully immunised. The proposed target for immunisation coverage in New Zealand was for 95% of children to have completed age-appropriate immunisation by two years of age, with this target to be reached by July 2012. The most recent data regarding this target showed 90% total immunisation coverage at two years (released by the Ministry of Health in September 2012). However substantial disparities exist between Māori and non-Māori in terms of the numbers of children immunised in a timely manner, particularly at six months of age [17]. For example, in the six-month period ending May 2012, 78% of non-Māori children had completed their age appropriate vaccinations at six months of age compared to 57% of Māori children. Not having completed age appropriate vaccinations at six months of age leaves infants more vulnerable to infection and subsequent hospital admissions, including for respiratory diseases such as bronchiectasis and pertussis [10].

### Health and clinical information datasets in New Zealand

In New Zealand, the Ministry of Health is responsible for the oversight and funding of New Zealand’s twenty District Health Boards (DHBs). Selected clinical information is reported by DHBs to the Ministry of Health, and is collated into national datasets and overseen by the Information Group within the Ministry of Health. Among the wide range of health related registries and datasets held in New Zealand are: the National Immunisations Register (NIR), National Minimum Data Set (NMDS, covering hospital discharges), Mortality Collection, and National Maternity Collection (MAT) [18].

Clinical information that is not reported to the Ministry of Health (so does not appear in these national collections) is held locally within independent clinical information systems maintained within each DHB: for example the Perinatal Information Management System (PIMS), which collects information on perinatal events.

### National health index number – the dataset linking key

A unique identification number - National Health Index number (NHI number) - is assigned to each person using health and disability support services. Approximately 95% of New Zealand citizens have a unique NHI number [19]. The NHI is an index of information associated with that unique number. The Health Information Privacy Code 1994 places restrictions on the creation and use of unique identifiers such as the NHI number [19].

The NHI register holds the following information: name (including alternative names such as maiden names), NHI number, address, date of birth, sex, New Zealand resident status, ethnicity, date of death, and flags indicating any medical warnings or donor information [19]. The NHI number enables the positive and unique identification of individuals for the purposes of treatment and care, and for maintaining medical records at both the primary and secondary sector care levels. As such, NHI numbers also allow researchers to link and analyse routinely collected health datasets to answer clinical questions, and to explore associations between a range of risk factors, exposures and health outcomes.

## Methods/Design

A retrospective cohort study comprising 15 years of maternal and infant data matched to local and national health information datasets is being used to assess a range of maternal and infant outcomes. Differences in outcome measures will be analysed to identify possible explanatory factors such as maternal age, parity, socioeconomic status and smoking, and to account for the degree to which ethnic disparities in infant health outcomes are mediated by these factors.

### Outcome measures

#### Primary outcomes

1. Rates of respiratory hospitalisations of infants (within one year of birth) calculated for infants of both Māori and non-Māori women (for mothers under 20 years of age, and overall) accounting for relationship to parity, maternal age, neighbourhood (small area) socioeconomic deprivation (NZDep Index [20,21]), maternal smoking status (recorded at time of antenatal booking in CCDHB system) and other risk factors (e.g. maternal age, maternal smoking status, socioeconomic status).

2. Proportion of infants with age appropriate immunisations at six months and one year, calculated for both infants born to Māori women and infants born to non-Māori women, and relationship to parity, maternal age, neighbourhood socioeconomic deprivation, smoking status, and other risk factors. As the National Immunisation Registry was not in place for the entire study period, this analysis is limited to a three year period between 1 July 2006 to 31 December 2009.

#### Secondary outcomes

1. Rates of all admissions to hospital of Māori women and infants compared to rates of admission for non-Māori women and infants in the first post natal year.

2. Pregnancy outcomes including infant peri-natal deaths and admissions to neonatal units, summarised and compared for Māori and non-Māori mothers.

3. Other pregnancy outcomes including number of postnatal visits, length of hospital stay following delivery, obstetric procedures, obstetric complications, outcome for babies, for example the infant’s Apgar score after 5 minutes and birth weight.

4. Proportion of mothers reporting fully breast feeding at hospital discharge for Māori compared to non-Māori.

### Study population and overview

The study population included pregnant women who gave birth in hospital in the Capital and Coast DHB (CCDHB) Wellington region between 1995–2009 and their infants (up to 1 year of age). Exclusion criteria include: babies born prior to 20 weeks gestation, babies born at home (planned or unplanned) and who were not subsequently admitted to hospital.

Ethnicity information for the mother was obtained from the self-reported measure obtained from the most recent health system contact. Infant ethnicity information was obtained from the hospital birth record. Up to three ethnicities are recorded for an individual, these were prioritised to obtain a single ethnicity for each mother/infant, in line with Ministry of Health protocols [22].

### Sample size calculation

Sample size calculations were based on nationally collected hospitalisation data 2003–2005 for respiratory hospitalisation rates [7]. For infants of less than one year of age these are just under 20,000 per 100,000 for Māori and just under 10,000 per 100,000 for non-Māori. Based on these respiratory hospitalisation rates for Māori and non-Māori infants in the first year of life, to detect a difference between a 200 events per 1000 infants of young Māori women (20% rate), and a 100 events per 1000 infants of young non-Māori group (10% rate) a sample of 316 women per ethnic group would be required (alpha = 0.05; power = 0.9). Focusing on outcomes for young mothers there have been infants born to 646 Māori women under the age of 20 years, and 1,002 non- Māori women under the age of 20 years, over the past ten years (1998–2007) in the CCDHB region (statistics supplied by CCDHB). The retrospective study will therefore have sufficient power to detect relatively small differences in rates between the Māori and non-Māori groups in this younger age bracket.

### Data collection and matching

**Figure 1:**
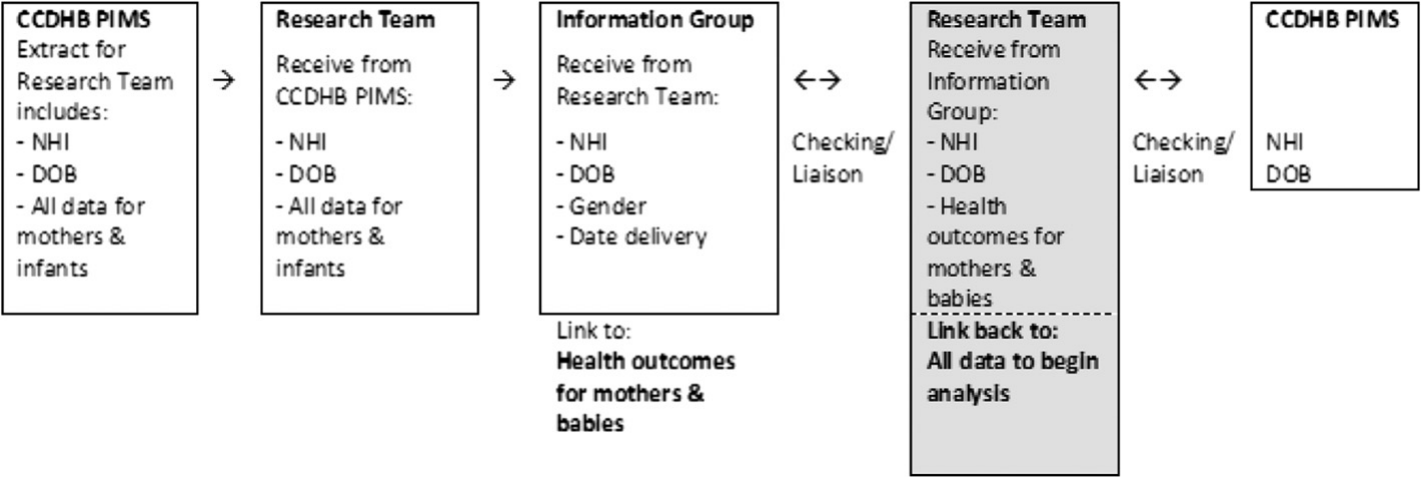
Overview of the information flow between the research team and Information Group.

The study uses routinely collected local hospital data from the CCDHB PIMS dataset (Table 1) linked (via NHI number) to national health datasets (Figure 1).

**Table 1:** Outline of data extracted from the CCDHB Perinatal Information Management System (PIMS)

**Information**	**Included fields**
Patient	NHI, DOB, domicile, marital status, ethnicity, birth country, education, registration with a Lead Maternity Carer (LMC), date of hospital admission
Pregnancy	Parity, gravida, smoking, alcohol, anaesthetics, scan history
Mortality	Place, date, cause
Antenatal	Follow up required (type and number)
Admissions	Admitted from, date
Care transfer	LMC transfer, reason
Discharge	Discharge date, from, to, reasons, length of stay
Ward transfer	Transfers from, to, dates, reasons,
Previous obstetric history	Previous birthweights, outcomes
Infant	Gestation, birth weight, Apgar score, birth order

**Figure 2:**
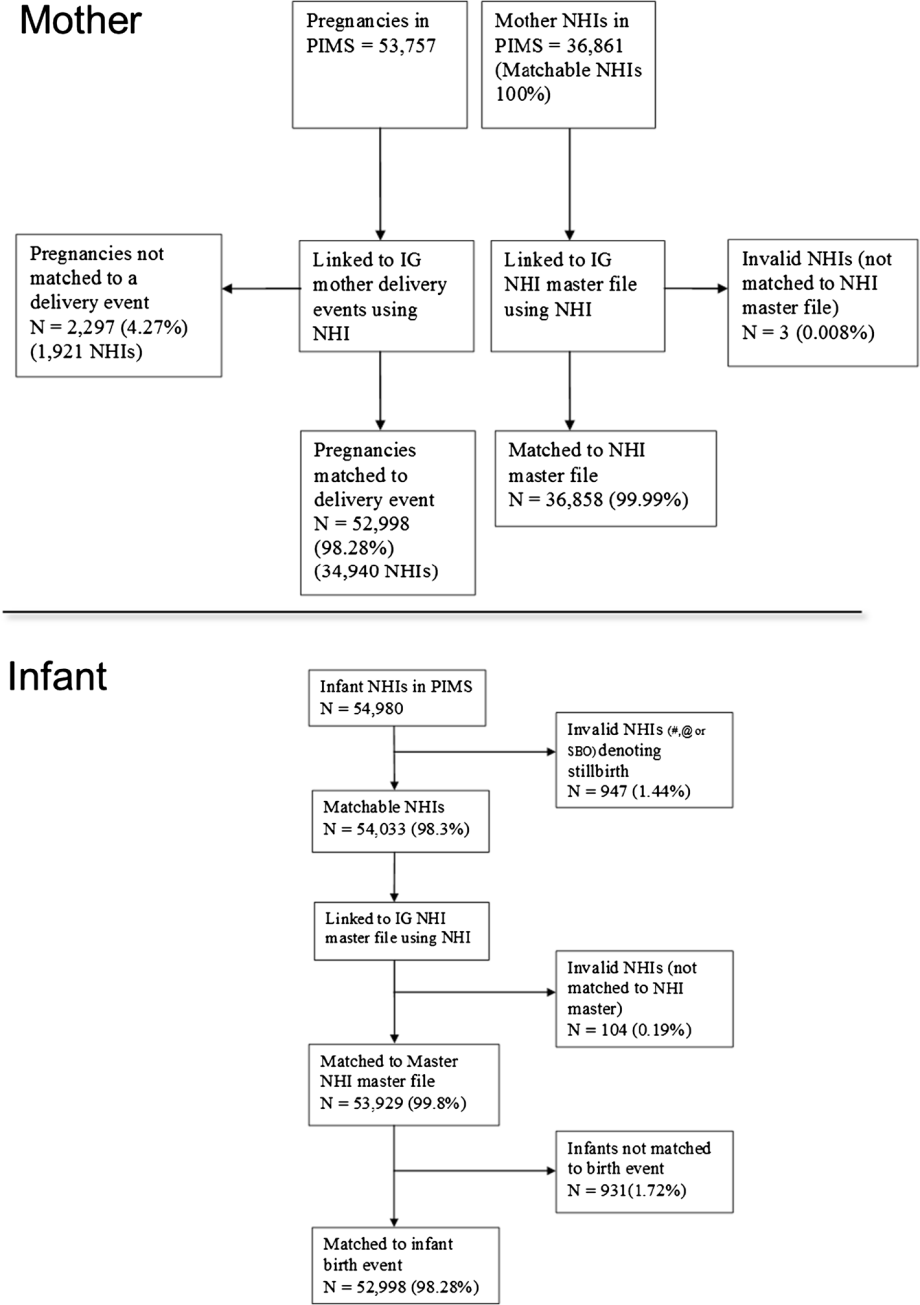
Matching rates between the routinely collected hospital data from the Capital and Coast District Health board (CCDHB) Perinatal Information Management System (PIMS) dataset and the national health information datasets held within the Information Group (IG) at the Ministry of Health.

Women were identified by NHI number and linked to their infant(s) using maternal NHI numbers. No personally identifiable data (i.e. names, addresses) were extracted from the PIMS database. Maternal and infant NHI numbers, alongside sex, date of birth (mothers) and date of delivery (infants), were sent to the Information Group at the Ministry of Health, who matched information on maternal and infant health outcomes from national data collections as outlined in Table 2 and Figure 1. These extracted data were then matched by the study team to the PIMS data to allow for analysis of primary and secondary outcomes. The matching rate from PIMS to the national datasets held within the Information Group for the mothers and their infants is shown in Figure 2. The matching rate of pregnancy information for the mother from the PIMS dataset to the mothers’ nationally held delivery event information is 98.28%, representing 34,940 individual women in the cohort, with 52,998 delivery/birth events. There were 54,980 individual infant NHI numbers held within PIMS datasets, with 98.28 % matched to an infant’s birth event (N = 52,998).

**Table 2:** Nationally held health information datasets used for matching to PIMS extracted data

**Health information dataset**	**Type of information held in dataset**	**Specific information extracted and matched**
Mortality Collection	Death	Date of death, gestation, birth-weight, diagnostic codes on cause of death
National Immunisation Register	Immunisation information	Immunisation at 6 and 12 months, registration status (active or not)
National Maternity Collection	Information on maternal and infant health collected by the lead maternal carer	Breastfeeding at 2 weeks and at transfer to well child provider, neonatal death, number of inpatient postnatal visits, number of midwife home visits
National Minimum Dataset (NMDS)	Hospital events	Maternal and/or infant hospital admissions (public) /discharge date, duration, diagnostic (ICD) codes
NMDS	Infant birth outcomes	Apgar at 5 minutes, birth weight dianostic codes, length of stay,
NMDS	Referrals	Date, specialist, provider, reason(s)

### Statistical analysis

Hospital admissions with respiratory related disease as the primary reason for admission were identified from the clinical codes (ICDs) and defined according to Craig et al., (2009) [10]. For the purpose of this study, each admission was treated as a separate event and the number of respiratory events per infant calculated. A multivariable Poisson regression model will be used to investigate how the rate of presentation for respiratory disease differs by ethnicity (Māori compared to non-Māori) and how it is influenced by potential risk factors (e.g. maternal age, maternal smoking status, socioeconomic status). The rates, rate ratios and their respective 95% confidence intervals will be reported for all outcomes.

### Ethical approval

Ethical approval for the study was granted by the New Zealand Central Region Ethics Committee (Ref CEN 09/09/068, 19 October 2009).

## Discussion

The analysis for this study is still underway. It is expected that this in-depth investigation from a mother to infant perspective will provide a greater understanding of the causes of health inequalities that exist between the infants of Māori and the infants of non-Māori. The methodology described in this paper could also be extended to include other health issues such as mental health. It is hoped that the results will inform the development of targeted interventions aimed at reducing postnatal hospital admissions for New Zealand’s infants.

## Competing interests

The authors declare they have no competing interests.

## Authors’ contributions

SF drafted the manuscript and is involved in the data analysis and interpretation of the data. SG is responsible for the acquisition of data and data cleaning, database management and interpretation of the data. JS is responsible for the statistical analysis and was involved in the design of the study. SR participated in the design of the study and help draft the manuscript. BR participated in the design of the study and help draft the manuscript. RE participated in the design of the study and its coordination. BL conceived of the study, participated in its design and has overall responsibility for the study. All authors read and approved the final manuscript.

## Pre-publication history

The pre-publication history for this paper can be accessed here:

http://www.biomedcentral.com/1471-2393/13/145/prepub
